# Cavernous hemangioma of the thymus: A case report and review of the literature

**DOI:** 10.1097/MD.0000000000011698

**Published:** 2018-07-27

**Authors:** Chunhui Zheng, Fangbiao Zhang, Shaosong Tu, Xiangyan Zhang, Chun Zhao

**Affiliations:** Department of Cardiothoracic Surgery, Zhejiang University, Lishui Hospital, Lishui Central Hospital, Lishui, Zhejiang, People's Republic of China.

**Keywords:** cavernous hemangioma, surgery, thymus

## Abstract

**Rationale:**

Cavernous hemangioma is a congenital venous malformation with the potential to develop in all tissues of the body. However, cavernous hemangioma of the thymus is extremely rare.

**Patient concerns:**

The present study describes the case of an asymptomatic, 30-year-old female who presented with a cavernous hemangioma in the thymus during a physical examination. Enhanced computed tomography of the chest revealed a 2.3 × 1.7 × 1.3 cm mass in the thymus.

**Diagnoses:**

Histopathological examination revealed that the tumor exhibited the typical histological findings of a cavernous hemangioma.

**Interventions:**

The patient underwent surgical resection due to the uncertain diagnosis and the possibility that the mass was a thymoma or teratoma.

**Outcomes:**

One-year post surgery, the patient was alive with no evidence of tumor recurrence.

**Lessons:**

Cavernous hemangioma of the thymus is a very rare disease. Complete surgical resection may be a critical therapeutic option.

## 1 Introduction

Cavernous hemangioma is a congenital venous malformation, which was initially described by Lungenschmid in 1990.^[1]^ In most studies of mediastinal tumors, the incidence of hemangioma, including cavernous hemangiomas, capillary hemangioma and mixed hemangioma, is 0.5% or less.^[2]^ The majority of cavernous hemangiomas occur superficially in the cutaneous and mucosal tissues of the face, mouth, and limbs particularly in children. In the early stages, patients often do not exhibit obvious clinical symptoms. As the tumor grows, patients present with oppressive symptoms that are attributed to the large size. Imaging examination particularly enhanced computed tomography (CT), aids in the determination of the location and size of the tumor and whether the mass is benign or malignant. Given the rarity of the condition, therapeutic strategies remain unclear. However, according to the published literature, complete resection is considered the most successful and effective treatment.^[3]^ The current study presents a rare case of cavernous hemangioma of the thymus in a 30-year-old woman and reviews the previously reported cases in the literature.

### 1.1 Case presentation

**Figure 1:**
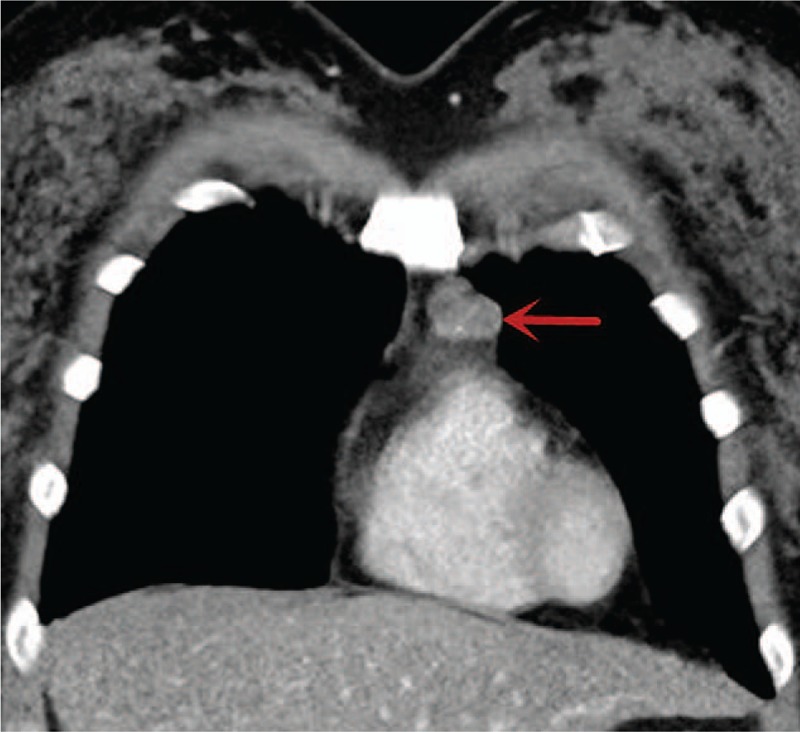
A chest CT scan revealing an oval soft tissue mass measuring 2 × 1.7 cm in size (arrow).

**Figure 2:**
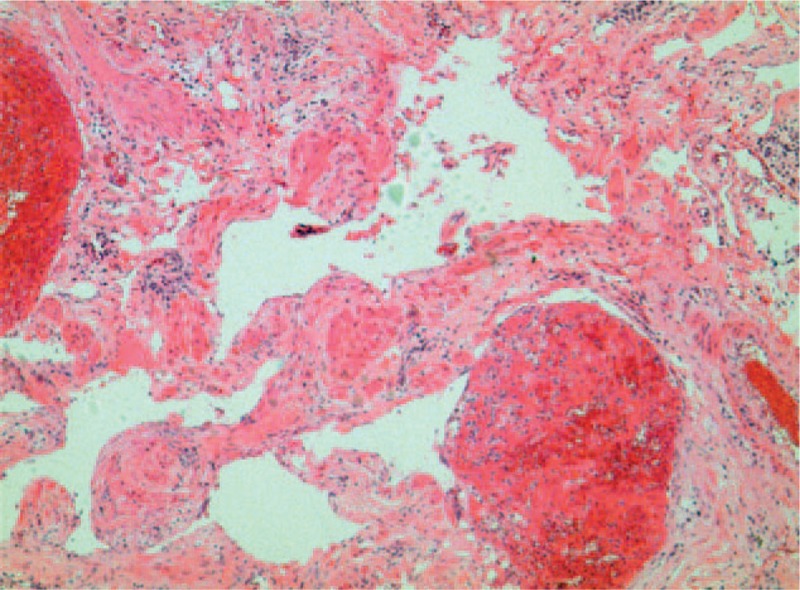
The hematoxylin-eosin stained cavernous hemangioma exhibits prominent ectatic vessels filled with blood (×10).

A 30-year-old, asymptomatic female presented to our hospital for a physical examination. The patient had a history of diabetes mellitus and no history of cigarette smoking, hepatitis, tuberculosis, hypertensive disease, or coronary disease. Written informed consent was obtained from the patient for the publication of the present study. Enhanced CT (Philips, Brilliance ICT CP 200063) of the chest revealed an anterior mediastinal oval tumor 2.3 × 1.7 × 1.3 cm in size with border regularity and without necrosis and calcification (Fig. 1). A preoperative diagnosis of thymoma was considered due to the enhanced CT features. Routine blood, coagulation function, liver function, serum electrolyte, and electrocardiogram results were all within normal limits (Table 1). For the purpose of providing a definitive diagnosis and treatment for an anterior mediastinal tumor such as thymoma, video-assisted thoracoscopic surgery (VATS) was performed under general anesthesia. Histopathological examinations using hematoxylin and eosin staining (Sinopharm Chemical Reagent Co., Ltd., Shanghai, China) revealed that the tumor exhibited the typical histological findings of a cavernous hemangioma, as it was comprised of a proliferation of sized vessels (Fig. 2). The patient presented with chylothorax on the second postoperative day and was discharged on the 13th postoperative day. One-year post surgery, the patient was alive with no evidence of tumor recurrence.

**Table 1:**
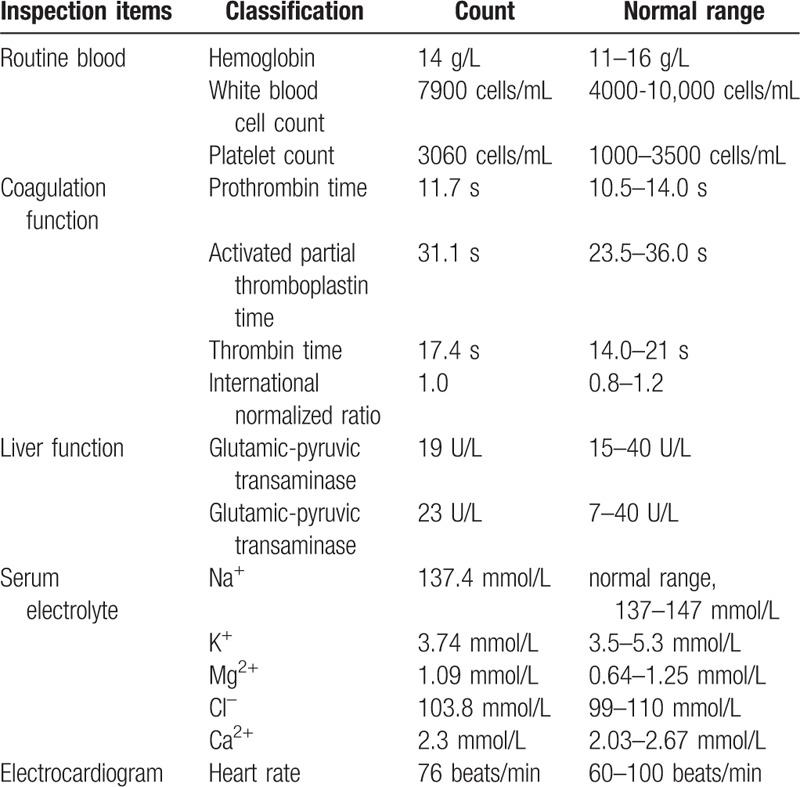
Patient examinations in our study.

### 1.2 Literature review

Relevant literature and studies regarding the cavernous hemangioma were searched in databases from January 1990 to January 2018. The text words and Mesh terms “cavernous hemangioma,” “thymus,” and “thymic” were used. Nine articles were identified from the database searches, and a total of 9 patients were included (Table 2).^[1,3–10]^ The patients included 5 males and 4 females. The mean age was 47.9 years, ranging from 27 to 71 years. All patients underwent surgery and recovered from the surgical procedure uneventfully. Seven patients underwent follow-up, and the mean length of the postoperative follow-up was 31.3 months (5–96 mos). One patient died.

**Table 2:**
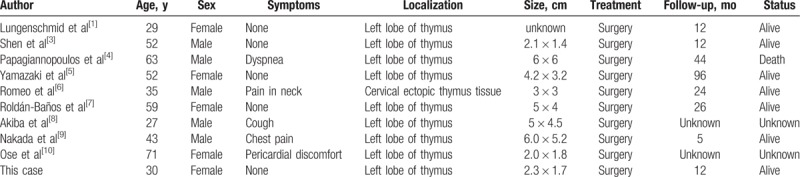
Characteristics of patients in published English literature.

## 2 Discussion

Cavernous hemangioma is a congenital venous malformation that occurs throughout the entire body but is rarely localized to the thymus. A large review of 1064 cases of mediastinal masses collected over a 40-year period was reported by Wychulis et al.^[11]^ In his report, only 5 cases were mediastinal hemangiomas, but none of the lesions were of thymic origin.^[11]^ Histologically, hemangioma originates from residual embryonic vascular cells and is caused by abnormal vascular development at the embryonic stage. In total, 90% of the hemangiomas are cavernous hemangioma and capillary hemangioma. As age increases, some of the lesions disappear without treatment. To the best of our knowledge, nine cases of thymic cavernous hemangioma have been reported. When the tumor is small, patients often present with no clear clinical symptoms. As the tumor gradually grows, patients present with different symptoms, such as chest pain, cough and dyspnea. Recurrent bleeding and cystic changes may cause rapid tumor growth. Reviewing the reported literature, 4 patients were asymptomatic, and the tumors were discovered incidentally. One, one, and two patients presented with cough, pericardial discomfort, and chest pain, respectively.

A thymic cavernous hemangioma is difficult to distinguish from another type of mediastinal tumor, such as a thymoma or teratoma. The differential diagnosis of a thymic cavernous hemangioma includes numerous malignant and benign tumors, including thymoma, lymphoma, teratoma, neuroma, angiolipoma, goiter, and cystic lymphangioma. Shen et al^[3]^ reported a case of cavernous hemangioma of the thymus misdiagnosed as thymoma. It is not possible to determine whether a mass is benign or malignant using imaging examinations, including chest CT and magnetic resonance imaging (MRI). However, imaging examinations are beneficial for assessing the tumor size, border and vascular condition. A correct diagnosis of a cavernous hemangioma can be made using several CT features, such as multiple venous lakes, calcified phleboliths, complex multiple venous channels, distant feeding veins, and delayed enhancement.^[9]^ Calcified phlebolith is the characteristic manifestation of cavernous hemangioma of the thymus. However, calcified phlebolith is observed in only 10% of the cases.^[9]^ Biopsies may not be suitable for definitive diagnosis due to bleeding.^[9]^ Therefore, surgical resection should be performed for a definitive diagnosis and treatment.^[9]^ Histopathology is significant for differentiating between these tumors. Histopathologically, a cavernous hemangioma is composed of dilated vessels covered by one layer of endothelial cells.^[3]^

The choice of treatment is dependent on the related organ and can include sclerotherapy, embolization, and surgical resection. Reviewing previously reported cases in the literature, complete surgical resection is generally accepted as the definitive and effective treatment of choice for thymic cavernous hemangioma. Median sternotomy or thoracotomy could be suitable for cases with a giant tumor or innominate vein aneurism.^[10]^ On the other hand, with the development of minimally invasive technology, most small and noninfiltrative thymic tumors could be safely and availably excised via video-assisted thoracoscopic surgery (VATS). Shen et al^[3]^ described the case of a 52-year-old male who underwent thymectomy using VATS. He considered thymectomy necessary to extirpate the lesion completely and that extirpation by VATS might be a better option to treat this condition compared with median sternotomy.^[3]^ In our study, the patient underwent VATS. The tumor should be completely removed during the operation, and attention should be paid to bleeding. Some reports suggest that the tumor recurs after incomplete resection or biopsy.^[2]^

## 3 Conclusion

We reported a rare case of thymic cavernous hemangioma that was definitively diagnosed after surgery, and we reviewed the previously reported cases in the literature. This study revealed three important findings. First, thymic cavernous hemangioma can occur at all ages and exhibits no sex predominance. Second, these tumors are often discovered incidentally. Finally, complete surgical resection presents a safe and effective treatment.

## Author contributions

Author ZCH drafted the article. ZC, ZXY, and ZFB performed the surgery. ZC and TSS helped collect clinical data and made critical revisions for important intellectual content. All authors read and approved the final article.

**Data curation:** Shaosong Tu.

**Project administration:** Xiangyan Zhang.

**Writing – original draft:** Chunhui Zheng.

**Writing – review & editing:** Fangbiao Zhang, chun zhao.
